# Optimal Cut-Offs of Homeostasis Model Assessment of Insulin Resistance (HOMA-IR) to Identify Dysglycemia and Type 2 Diabetes Mellitus: A 15-Year Prospective Study in Chinese

**DOI:** 10.1371/journal.pone.0163424

**Published:** 2016-09-22

**Authors:** C. H. Lee, A. Z. L. Shih, Y. C. Woo, C. H. Y. Fong, O. Y. Leung, E. Janus, B. M. Y. Cheung, K. S. L. Lam

**Affiliations:** 1 Department of Medicine, The University of Hong Kong, Hong Kong S.A.R., China; 2 Department of Medicine, University of Melbourne and Western Health, Melbourne, Victoria, Australia; 3 Research Center of Heart, Brain, Hormone and Healthy Aging, The University of Hong Kong, Hong Kong S.A.R., China; 4 State Key Laboratory of Pharmaceutical Biotechnology, The University of Hong Kong, Hong Kong S.A.R., China; Shanghai Diabetes Institute, CHINA

## Abstract

**Background:**

The optimal reference range of homeostasis model assessment of insulin resistance (HOMA-IR) in normal Chinese population has not been clearly defined. Here we address this issue using the Hong Kong Cardiovascular Risk Factor Prevalence Study (CRISPS), a prospective population-based cohort study with long-term follow-up.

**Material & Methods:**

In this study, normal glucose tolerance (NGT), impaired fasting glucose (IFG), impaired glucose tolerance (IGT) and type 2 diabetes mellitus (T2DM) were defined according to the 1998 World Health Organization criteria. Dysglycemia referred to IFG, IGT or T2DM. This study comprised two parts. Part one was a cross-sectional study involving 2,649 Hong Kong Chinese subjects, aged 25–74 years, at baseline CRISPS-1 (1995–1996). The optimal HOMA-IR cut-offs for dysglycemia and T2DM were determined by the receiver-operating characteristic (ROC) curve. Part two was a prospective study involving 872 subjects who had persistent NGT at CRISPS-4 (2010–2012) after 15 years of follow-up.

**Results:**

At baseline, the optimal HOMA-IR cut-offs to identify dysglyceia and T2DM were 1.37 (AUC = 0.735; 95% confidence interval [CI] = 0.713–0.758; Sensitivity [Se] = 65.6%, Specificity [Sp] = 71.3%] and 1.97 (AUC = 0.807; 95% CI = 0.777–0.886; Se = 65.5%, Sp = 82.9%) respectively. These cut-offs, derived from the cross-sectional study at baseline, corresponded closely to the 75^th^ (1.44) and 90^th^ (2.03) percentiles, respectively, of the HOMA-IR reference range derived from the prospective study of subjects with persistent NGT.

**Conclusions:**

HOMA-IR cut-offs, of 1.4 and 2.0, which discriminated dysglycemia and T2DM respectively from NGT in Southern Chinese, can be usefully employed as references in clinical research involving the assessment of insulin resistance.

## Introduction

Insulin resistance mediates a number of cardio-metabolic disorders, and plays a crucial role in the pathogenesis of the metabolic syndrome [1]. Dysglycemia, on the other hand, is a major clinical manifestation of insulin resistance. Blood glucose increases when there is insufficient insulin to overcome the resistance to its metabolic actions in maintaining glucose homeostasis. Over the years, there have been a number of methods to assess insulin resistance [2]. The hyperinsulinemic euglycemic glucose clamp, albeit the gold standard technique, is limited by its complexity, invasiveness, high cost and laborious requirements. The homeostasis model assessment of insulin resistance (HOMA-IR), on the contrary, provides a convenient and inexpensive means of estimating insulin resistance, and the estimates derived have been shown to correlate well with those derived from the euglycemic clamp [3, 4]. Evaluation of insulin resistance is useful in clinical practice, and even more importantly, in diabetes and metabolic research. For instance, HOMA-IR had been utilized to identify eligible subjects with insulin resistance in a recently published, multi-center, randomized, placebo-controlled trial that assessed the effect of pioglitazone in secondary prevention of cardiovascular events [5].

As in any clinical test, a reference range or cut-off is necessary for discrimination of a disease from normal physiological conditions. The normal reference range of HOMA-IR in Chinese population has however not been clearly defined. Certainly, ethnic differences in HOMR-IR cut-offs exist across different populations [6–9]. In addition, most of these cut-offs were derived from cross-sectional studies that could be confounded by various limitations. In most studies, HOMR-IR cut-offs were derived either from the lower boundary of the top quintile [10], or the 75^th^ or 90^th^ percentiles of HOMR-IR estimates from individuals with normal body weight and glycemia without metabolic disorders evaluated at a single time point [11–13]. Notably, glycemic status of an individual may change over time. In the Diabetes Prevention Program Outcomes Study [14], 10 and 13% of subjects with pre-diabetes reverted back to normoglycemia over 5.7 years in control and lifestyle intervention group, respectively. On the other hand, we previously also reported that 16% of subjects with normal glucose tolerance at baseline could have deterioration of glycemia over a period of 5.4 years [15]. Taken together, HOMA-IR reference range or cut-offs obtained from cross-sectional studies can be inaccurate and limited by the inclusion of a proportion of subjects whose glycemic status may either deteriorate or improve after the initial evaluation. We therefore performed this study, using both cross-sectional and prospective approaches, to establish optimal HOMA-IR cut-offs for identifying dysglycemia and type 2 diabetes mellitus (T2DM), and a normal reference range of HOMA-IR in Southern Chinese respectively, based on the Hong Kong Cardiovascular Risk Factor Prevalence Study (CRISPS), a population-based prospective cohort study with 15 years of follow up, including subjects with normoglycemia throughout this time.

## Materials & Methods

### Study Patients

This current study involved subjects from CRISPS, a population-based and long-term prospective study on the development of cardiovascular risk factors in Hong Kong. The study protocol was approved by the institutional review board of the University of Hong Kong / Hospital Authority Hong Kong West Cluster. All subjects gave written informed consent prior to any study related procedures.

Briefly, 2,895 unrelated Hong Kong Chinese subjects aged between 25 and 74 years were recruited in CRISPS-1 (1995–1996) through randomly selected telephone numbers and detailed metabolic risk assessments were performed at baseline. Subjects were followed up in CRISPS-2 (2000–2004), CRISPS-3 (2005–2008) and CRISP-4 (2010–2012). Details of medical history taking, anthropometric and biochemical parameters were described previously [16]. Central obesity was defined as having a waist circumference (WC) of ≥90 cm in men and ≥80 cm in women. Hypertension was defined as blood pressure ≥140/90mmHg or on anti-hypertensive medications. Dyslipidemia was defined as having high fasting triglycerides ≥1.69 mmol/L, low HDL-C <1.04 mmol/L in men or <1.29 mmol/L in women, and high LDL-C ≥ 3.4 mmol/L or taking lipid lowering agents.

All subjects, except those taking anti-diabetic medications for known T2DM diagnosed by their family physicians, had a 75g oral glucose tolerance test (OGTT) when they attended assessment from CRISPS-1 to CRISPS-4. Subjects were categorized as having normal glucose tolerance (NGT), impaired fasting glucose (IFG), impaired glucose tolerance (IGT), or T2DM according to the World Health Organization (WHO) 1998 diagnostic criteria: IFG = fasting glucose (FG) ≥ 6.1 mmol/L and <7.0 mmol/L; IGT = FG <7.0 mmol/L and 2-hour post OGTT glucose (2hG) ≥ 7.8 mmol/L and <11.1 mmol/L; T2DM = FG ≥7.0 mmol/L or 2hG ≥ 11.1 mmol/L [17]. In our study, dysglycemia referred to IFG, IGT or T2DM, and non-DM referred to NGT, IFG or IGT. Insulin was measured by micro-particle enzyme immunoassay (Abbott Laboratories, Tokyo, Japan), and HOMR-IR was calculated using a mathematical formula as follows [18]:

HOMA-IR = (fasting insulin [μlU/ml] x fasting glucose [mmol/L]) / 22.5

### Study Design

This study comprised two parts. Part one was a cross-sectional study to determine the optimal HOMA-IR cut-offs for the discrimination of dysglycemia from NGT, and T2DM from non-DM, respectively. All subjects from CRISPS-1, who had valid baseline HOMA-IR values, were included for analysis. Part two was a prospective study to evaluate the optimal reference range of HOMA-IR in the normal Chinese population, based on a subgroup of subjects with persistent NGT, which was defined as having NGT at both CRISPS-1 and CRISPS-4, and not identified with dysglycemia at any point during the 15 years of prospective follow-up.

### Statistical Analysis

Data were analyzed using IBM SPSS statistics 23.0. Normally distributed data were presented as means ± standard deviation (SD). Non-normally distributed data were transformed to normality by natural logarithm prior to analysis and presented as medians (inter-quartile range). Changes in cardiovascular risk profiles of subjects were compared with successive HOMA-IR quartiles (p for trend) using one-way ANOVA for continuous variables or gamma for ordinal variables as appropriate. Pearson's correlation coefficient (r) was calculated to determine the strength of association between groups. In the first part of this study, at baseline, the optimal HOMA-IR cut-offs to discriminate dysglycemia from NGT, and T2DM from non-DM, respectively, were determined as the point with the maximum Youden index (J) on the receiver-operating characteristic operation (ROC) curve with J = sensitivity + specificity—1. The total area under the ROC curve (AUROC) with 95% confidence interval (CI), sensitivity, specificity, positive predictive value (PPV) and negative predictive value (NPV) of HOMA-IR values to discriminate dysglycemia from NGT, and T2DM from non-DM, respectively, were also calculated. In all analyses, statistical significance was achieved at p < 0.05.

## Results

### Part One: Cross-Sectional Analysis

In the first part of this study, a total of 2,649 subjects from CRISPS-1 were included in the cross-sectional analysis, after exclusion of 246 subjects without complete data or valid HOMA-IR values. Table 1 shows the demographic and metabolic parameters of subjects based on their glycemic status (NGT versus dysglycemia). At baseline, the median HOMA-IR of subjects with NGT was 1.00 (0.66–1.48), while those with dysglycemia had a significantly higher HOMA-IR of 1.78 (1.06–2.73) (p <0.001). Compared with those with NGT, subjects with dysglycemia were older, less likely to be current smokers, more obese with central obesity, more hypertension and more dyslipidemia (p <0.001).

**Table 1:** Descriptive statistics of subjects by baseline glycemic status at CRISPS1 (N = 2649). Data was present as mean±SD or median (interquartile range); *Log-transformed before analysis. BMI, body mass index; WC, waist circumference; WHR, waist-hip-ratio; SBP, systolic blood pressure; DBP, diastolic blood pressure; HT, hypertension; FG, fasting glucose; 2hG, 2-hour glucose post OGTT; HOMA-IR, Homeostasis Model Assessment-Insulin Resistance; NGT, normal glucose tolerance; T-Chol, total cholesterol; TG, triglycerides; HDL-C, high density lipoprotein cholesterol; LDL-C; low density lipoprotein cholesterol.

Variables	NGT	Dysglycemia	p-value
Number	1954	695	—
Age, years	43.3±11.9	53.0±12.4	<0.001
Gender, % women	49.6	53.2	0.057
Smoking (%)			0.009
Never smoke	74.5	74.5	—
Former smoker	5.9	8.9	—
Current smoker	19.6	16.5	—
			—
BMI, kg/m^2^	23.5±3.4	25.8±3.6	<0.001
WC, cm	77.3±9.5	84.5±10.1	<0.001
Central obesity, %	29.2	49.9	<0.001
WHR*	0.82 (0.77–0.88)	0.89 (0.83–0.94)	<0.001
			—
SBP, mmHg	116±17	130±23	<0.001
DBP, mmHg	73±10	79±12	<0.001
HT, %	11.4	38.3	<0.001
			—
FG, mmol/L	5.0±0.4	6.5±2.3	<0.001
2hG*, mmol/L	5.6 (4.8–6.4)	9.1 (8.3–11.6)	<0.001
Fasting insulin*, pmol/L	30.6 (20.8–45.8)	45.8 (29.2–68.8)	<0.001
HOMA-IR*	1.00 (0.66–1.48)	1.78 (1.06–2.73)	<0.001
			—
T-Chol, mmol/L	4.98±1.01	5.39±0.97	<0.001
TG*, mmol/L	0.90 (0.70–1.30)	1.30 (0.98–1.99)	<0.001
HDL-C, mmol/L	1.29±0.33	1.15±0.31	<0.001
LDL-C, mmol/L	3.18±0.86	3.50±0.87	<0.001
Dyslipidemia, %	58.0	81.3	<0.001

Pearson’s correlation analysis showed that at baseline, HOMA-IR positively correlated with age (r = 0.117), BMI (r = 0.470), WC (r = 0.445), SBP (r = 0.290), DBP (r = 0.274); fasting and 2-hour post OGTT plasma glucose concentration (r = 0.358 and 0.375, respectively), total cholesterol (r = 0.121), triglyceride (r = 0.348), LDL-C (r = 0.122), and negatively correlated with HDL-C (r = -0.265) (all p < 0.001). Table 2 presents the correlation of clinical and biochemical variables with HOMA-IR when baseline HOMA-IR values at CRISPS-1 were further categorized into quartile groups. Briefly, increasing HOMA-IR quartiles were associated with increasing age and obesity indices, higher blood pressure, greater lipid abnormalities and increasing glycemia.

**Table 2:** Correlation of baseline clinical and biochemical parameters with successive HOMA-IR quartiles at CRISPS1 (N = 2649). Data was present as means±SD or medians (interquartile range).

Variables	HOMA-IR				
	Q1: ≤0.72	Q2: 0.73–1.13	Q3: 1.14–1.83	Q4: ≥1.84	p for trend
Number	667	664	658	660	—
Gender, % of women	45.0	52.4	51.1	53.9	0.003
Age, years	44.5±12.7	44.5±12.5	45.9±12.5	48.5±12.9	<0.001
Ever smoke, %	33.4	25.4	20.9	22.1	<0.001†
BMI, kg/m^2^	22.2±2.9	23.1±3.0	24.6±3.1	26.7±3.6	<0.001
WC, cm	74.0	76.3	80.4	86.2	<0.001
Central obesity, %	20.2	24.7	40.9	53.0	<0.001†
WHR*	0.80 (0.76–0.86)	0.82 (0.77–0.88)	0.85 (0.79–0.90)	0.88 (0.82–0.93)	<0.001
					<0.001
SBP, mmHg	114±18	115±17	120±19	129±21	<0.001
DBP, mmHg	72±10	72±10	76±10	80±11	<0.001
HT (%)	9.7	9.9	18.1	36.1	<0.001†
FG, mmol/L	4.9±0.7	5.1±0.6	5.3±0.8	6.3±2.3	<0.001
2hG*, mmol/L	5.4 (4.5–6.5)	5.7 (4.9–6.9)	6.2 (5.3–7.5)	7.4 (6.0–9.7)	<0.001
Fasting insulin*, pmol/L	16.7 (13.2–20.1)	28.5 (25.7–31.9)	42.4 (38.2–47.9)	70.1 (59.7–88.9)	<0.001
DM, %	2.4	3.3	7.3	27.0	<0.001†
T-Chol, mmol/L	5.00±1.07	4.94±1.03	5.11±0.94	5.30±0.97	<0.001
TG*, mmol/L	0.80 (0.60–1.16)	0.90 (0.62–1.24)	1.10 (0.80–1.50)	1.36 (1.00–2.00)	<0.001
HDL-C, mmol/L	1.36±0.34	1.29±0.33	1.22±0.30	1.13±0.30	<0.001
LDL-C, mmol/L	3.16±0.86	3.14±0.89	3.32±0.84	3.43±0.87	<0.001
Dyslipidemia, %	49.8	57.2	69.1	80.6	<0.001†

†Ordinal gamma test

*Log-transformed before analysis. BMI, body mass index; WC, waist circumference; WHR, waist-hip-ratio; SBP, systolic blood pressure; DBP, diastolic blood pressure; HT, hypertension; FG, fasting glucose; 2hG, 2-hour glucose post OGTT; HOMA-IR, Homeostasis Model Assessment-Insulin Resistance; NGT, normal glucose tolerance; T-Chol, total cholesterol; TG, triglycerides; HDL-C, high density lipoprotein cholesterol; LDL-C; low density lipoprotein cholesterol.

**Fig 1:**
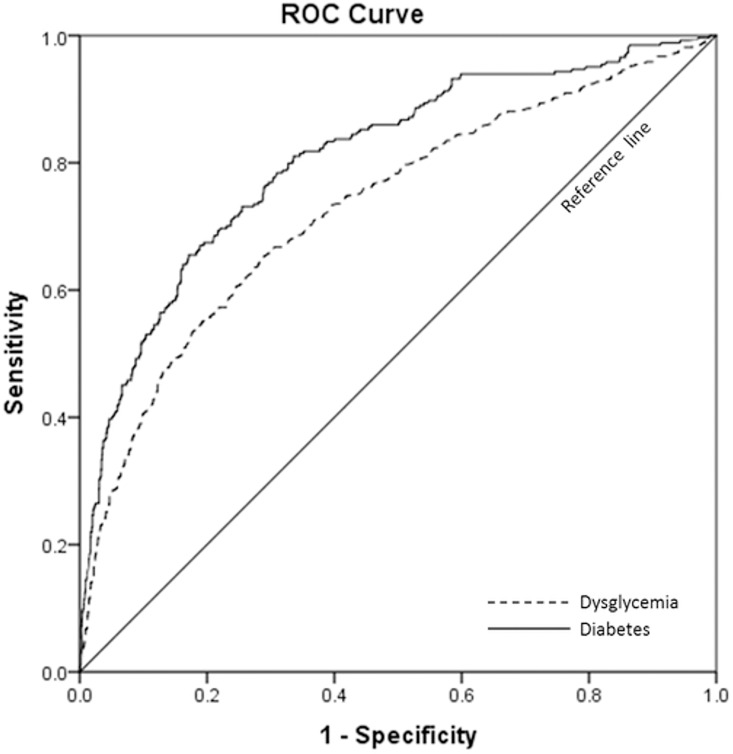
The receiver-operating characteristic curves of baseline HOMA-IR for detecting dysglycemia (dotted line) or type 2 diabetes (solid line).

The ROC curve of HOMA-IR values to distinguish dysglycemia from NGT, and T2DM from non-DM, respectively, at baseline is shown in Fig 1. The AUROC (95% CI) was 0.735 for dysglycemia and 0.807 for T2DM. Based on the maximum Youden index on the ROC curve, the optimal cut-off points of HOMA-IR to discriminate dysglycemia from NGT, and T2DM from non-DM, were 1.37 (Sensitivity 65.6% and specificity 71.3%) and 1.97 (Sensitivity 65.5% and specificity 82.9%) respectively (Table 3). If arbitrary cut-off values of 1.40 for dysglycemia and 2.0 for T2DM were adopted, which were also close to the optimal HOMA-IR cut-offs on ROC, the sensitivity were 69.1% for dysglycemia and 64.0% for T2DM, and the specificity were 75.8% for dysglycemia and 75.5% for T2DM. The PPV was 55.0% for dysglycemia and 30.3% for T2DM and the NPV was 85.1% for dysglycemia and 92.6% for T2DM (Table 3).

**Table 3:** Optimal cut‐off points for baseline HOMA-IR to discriminate dysglycemia from normal glucose tolerance, or type 2 diabetes from non-diabetes, at baseline (N = 2649).

	AUROC (95% CI)	Cutoff	Sensitivity, %	Specificity, %	PPV, %	NPV, %
Dysglycemia	0.735 (0.713–0.758)	1.37*	65.6	71.3	44.9	85.4
		**1.40†**	**69.1**	**75.8**	**55.0**	**85.1**
		1.50†	63.8	77.7	53.1	84.4
Diabetes	0.807 (0.777–0.886)	1.97*	65.5	82.9	29.8	95.6
		**2.0†**	**64.0**	**75.5**	**30.3**	**92.6**
		2.5†	48.9	91.2	38.1	94.2
		3.0†	39.8	95.3	48.4	93.5

*Optimal cut-offs by Youden j index

†Arbitrary value. AUROC, area under the curve of the Receiver Operating Characteristic; PPV, positive predictive value; NPV, negative predictive value.

### Part Two: Prospective Analysis

In the second part of our investigation, which was a prospective study to evaluate the optimal reference range of HOMA-IR in the normal Chinese population, a total of 872 subjects with persistent NGT after 15 years of follow-up were included for analysis. Baseline characteristics of these subjects at CRISPS1 are shown in S1 Table. Table 4 presents the HOMA-IR percentiles at baseline (CRISPS-1) of these subjects with persistent NGT on long-term follow-up. The 2.5^th^ and 95^th^ HOMA-IR percentiles of this persistent NGT group were 0.274 and 2.446. Importantly, in these 872 subjects who remained NGT after 15 years of prospective follow-up, the baseline HOMA-IR values at the 75^th^ and 90^th^ percentiles, the commonly used percentile thresholds to determine cut-off levels, were 1.440 and 2.028 respectively, and were strikingly similar to the respective optimal cut-offs for dysglycemia (1.4) and T2DM (2.0) obtained from the cross-sectional analysis of the data of 2,649 subjects at CRISPS-1.

**Table 4:** Reference range of HOMA-IR derived from the persistent NGT group* (N = 872).

Percentile	HOMA-IR
	Persistent NGT Group (N = 872)
2.5^th^	0.274
5^th^	0.343
10^th^	0.424
25^th^	0.645
50^th^, median	0.939
70^th^	1.288
71^st^	1.314
72^nd^	1.328
73^rd^	1.350
74^th^	1.391
75^th^	**1.440**
76^th^	1.458
80^th^	1.872
87^th^	1.872
90^th^	**2.028**
91^st^	2.085
95^th^	2.446

* Persistent NGT group refers to subjects with NGT at both CRISPS 1 and 4, and were not identified with DM at any point throughout the study.

## Discussion

In the present study, using two approaches, involving both cross-sectional and prospective analyses, we have derived optimal HOMA-IR cut-offs of 1.4 and 2.0, to discriminate dysglycemia from NGT, and T2DM from non-DM, respectively in Southern Chinese. This approach is different from the vast majority of studies that derived HOMA-IR cut-offs from cross-sectional cohorts at only a single time point.

In our study, HOMA-IR correlated with various metabolic parameters at baseline, including blood pressure, as previously reported [19, 20] As only 75 subjects were on anti-hypertensive medications, the relationship between HOMA-IR and the control of hypertension could not be assessed. Previous studies had shown significant ethnic differences in the HOMA-IR cut-offs between the Asian and the Western populations. While using the 90^th^ percentile of the non-obese NGT group as the HOMR-IR cut-off, two Japanese studies suggested the threshold values for insulin resistance were 1.7 [13], and 1.97 [12] respectively. In another recent Japanese study involving 2,153 healthy subjects, a reference range for HOMR-IR was established as between 0.4 and 2.4 [9]. Nonetheless, this reference range for Asians, derived from a cross-sectional study, could be confounded by limitations, since subjects in these cross-sectional cohorts could develop deterioration or improvement of glycemia over time. In contrast, the strengths of our study, which comprised both cross-sectional and prospective analyses of our cohort, together with the availability of OGTT data in subsequent clinical visits, enable the derivation of a normal reference range of HOMR-IR based on a super-control group who remained as persistent NGT after a prospective follow-up of 15 years.

First, in a cross-sectional analysis of more than 2,500 Chinese subjects at baseline, we identified optimal HOMA-IR cut-offs for discrimination of dysglycemia from NGT, and T2DM from non-DM, respectively. Next, from a group of subjects identified to have persistent NGT during 15 years of prospective follow-up, we defined a normal reference range of HOMA-IR in Chinese. Interestingly, the two HOMA-IR cut-offs, 1.4 for dysglycemia and 2.0 for T2DM, also fell onto the 75th and 90th percentile of HOMR-IR distribution values in the persistent NGT group, which are the commonly used reference points for definition of threshold cut-offs in previous studies on HOMA-IR [11–13]. Therefore, combining these two different approaches, we recommend the HOMA-IR cut-offs of 1.4 and 2.0 for discrimination of dysglycemia from NGT, and T2DM from non-DM, respectively in Chinese.

In the present study, dysglycemia and T2DM were chosen as the outcomes of interest. Firstly, these are the major clinical manifestations of insulin resistance. Furthermore, the associations between dysglycemia and cardiovascular outcomes, mortality and even cancer have been increasingly recognized [21–23]. The Emerging Risk Factors Collaboration had demonstrated the graded relationship between fasting glucose and cardiovascular outcome, with every 1 mmol/L rise in fasting glucose at above 5.6 mmol/L increasing the risk of coronary heart disease by 12% [21]. Similarly, every 1% increment of HbA1c levels had been shown to increase the risk of cardiovascular disease by at least 20% even in non-diabetic individuals [23]. Insulin resistance undoubtedly plays a crucial role in mediating the development of dysglycemia and T2DM. The derivation of optimal HOMA-IR cut-offs to discriminate dysglycemia from NGT, and T2DM from non-DM, respectively, in Chinese is useful not only in clinical practice, but more importantly, in diabetes and metabolic research on insulin resistance.

## Study Limitations

There were several limitations in our study. First, euglycemic clamp was not performed and hence there was no comparative gold standard evaluation of insulin resistance for the HOMA-IR estimates in our study, although previous studies had suggested a high correlation of insulin resistance values assessed by these two approaches [3]. Second, although HOMA-IR was evaluated in all subjects at each clinical assessment from CRISPS-1 to CRISPS-4, only a single glucose/insulin pair was performed at each clinical visit. Third, the diagnosis of T2DM was not sufficiently rigorous, being based on the use of anti-diabetic medications, or on the findings of only a single OGTT, the results of which are not always reproducible. For those on anti-diabetic medications, no information was available on the data upon which the diagnosis was based by their attending physicians. Lastly, HbA1c was not used for diagnosis of T2DM in our study, as it was not widely accepted as a diagnostic criterion until 2011 onwards [24].

Nonetheless, using this prospective cohort with 15 years of follow-up, our study had derived two HOMA-IR cut-offs, of 1.4 and 2.0, to discriminate dysglycemia from NGT, and T2DM from non-DM, respectively in Southern Chinese. These cut-off values can serve as useful references in clinical research involving the assessment of insulin resistance.

## Supporting Information

S1 Table Baseline characteristics of subjects with persistent normal glucose tolerance after 15 years of follow-up at CRISPS1 (N = 872). Data was present as mean±SD or median (interquartile range); *Log-transformed before analysis. BMI, body mass index; WC, waist circumference; WHR, waist-hip-ratio; SBP, systolic blood pressure; DBP, diastolic blood pressure; HT, hypertension; FG, fasting glucose; 2hG, 2-hour glucose post OGTT; HOMA-IR, Homeostasis Model Assessment-Insulin Resistance; NGT, normal glucose tolerance; T-Chol, total cholesterol; TG, triglycerides; HDL-C, high density lipoprotein cholesterol; LDL-C; low density lipoprotein cholesterol.
(DOCX)
