# Supplementary material for: Optimal Cut-Offs of Homeostasis Model Assessment of Insulin Resistance (HOMA-IR) to Identify Dysglycemia and Type 2 Diabetes Mellitus: A 15-Year Prospective Study in Chinese
Source: PLoS One. 2016 Sep 22;11(9):e0163424. doi: 10.1371/journal.pone.0163424 (PMC5033570; doi:10.1371/journal.pone.0163424)
Supplement: S1 Table — Data was present as mean±SD or median (interquartile range); *Log-transformed before analysis. BMI, body mass index; WC, waist circumference; WHR, waist-hip-ratio; SBP, systolic blood pressure; DBP, diastolic blood pressure; HT, hypertension; FG, fasting glucose; 2hG, 2-hour glucose post OGTT; HOMA-IR, Homeostasis Model Assessment-Insulin Resistance; NGT, normal glucose tolerance; T-Chol, total cholesterol; TG, triglycerides; HDL-C, high density lipoprotein cholesterol; LDL-C; low density lipoprotein cholesterol. (DOCX) [file pone.0163424.s001.docx]

Supplementary Table 1. Baseline characteristics of subjects with persistent normal glucose tolerance after 15 years of follow-up at CRISPS1 (N=872)

| Variables | All |
| --- | --- |
| Number | 872 |
| Age, years | 40.9±10.1 |
| Gender, % women | 54.3 |
| Smoking (%) |  |
| Never smoker | 79.0 |
| Former smoker | 4.3 |
| Current smoker | 16.8 |
|  |  |
| BMI, kg/m^2^ | 23.2±3.0 |
| WC, cm | 75.9±8.9 |
| Central obesity, % | 24.5 |
| WHR* | 0.81 (0.76-0.86) |
|  |  |
| SBP, mmHg | 113±15 |
| DBP, mmHg | 72±10 |
| HT, % | 7.3 |
|  |  |
| FG, mmol/L | 5.0±0.4 |
| 2hG*, mmol/L | 5.4 (4.7-6.2) |
| Fasting insulin*, pmol/L | 29.9 (20.8-39.6) |
| HOMA-IR* | 0.94 (0.64-1.44) |
|  |  |
| T-Chol, mmol/L | 4.89±1.06 |
| TG*, mmol/L | 0.82 (0.60-1.20) |
| HDL-C, mmol/L | 1.31±0.31 |
| LDL-C, mmol/L | 3.08±0.81 |
| Dyslipidemia, % | 54.8 |

Data was present as mean±SD or median (interquartile range); *Log-transformed before analysis.

BMI, body mass index; WC, waist circumference; WHR, waist-hip-ratio; SBP, systolic blood pressure; DBP, diastolic blood pressure; HT, hypertension; FG, fasting glucose; 2hG, 2-hour glucose post OGTT; HOMA-IR, Homeostasis Model Assessment-Insulin Resistance; NGT, normal glucose tolerance; T-Chol, total cholesterol; TG, triglycerides; HDL-C, high density lipoprotein cholesterol; LDL-C; low density lipoprotein cholesterol.
